# Clinical effectiveness of post-operative splinting after surgical release of Dupuytren's contracture: a systematic review

**DOI:** 10.1186/1471-2474-9-104

**Published:** 2008-07-21

**Authors:** Debbie Larson, Christina Jerosch-Herold

**Affiliations:** 1 Department of Occupational Therapy, Norfolk and Norwich University Hospital, NHS Foundation Trust, Norwich, UK; 2 School of Allied Health Professions, University of East Anglia, Norwich, UK

## Abstract

**Background:**

Splinting after contracture release for Dupuytren's disease of the hand is widely advocated. The purpose of this systematic review was to evaluate the quantity and quality of evidence regarding the effectiveness of splinting in the post-surgical management of Dupuytren's contractures.

**Methods:**

Studies were identified by searching the electronic databases Medline, AMED, CINAHL and EMBASE. Studies were included if they met the following inclusion criteria: prospective or retrospective, experimental, quasi-experimental or observational studies investigating the effectiveness of static or dynamic splints worn day and/or night-time for at least 6 weeks after surgery and reporting either individual joint or composite finger range of motion and/or hand function. The methodological quality of the selected articles was independently assessed by the two authors using the guidelines for evaluating the quality of intervention studies developed by McDermid.

**Results:**

Four studies, with sample sizes ranging from 23 to 268, met the inclusion criteria for the systematic review. Designs included retrospective case review, prospective observational and one controlled trial without randomisation. Interventions included dynamic and static splinting with a mean follow-up ranging from 9 weeks to 2 years. Pooling of results was not possible due to the heterogeneity of interventions (splint type, duration and wearing regimen) and the way outcomes were reported.

**Conclusion:**

There is empirical evidence to support the use of low load prolonged stretch through splinting after hand surgery and trauma, however only a few studies have investigated this specifically in Dupuytren's contracture. The low level evidence regarding the effect of post-operative static and dynamic splints on final extension deficit in severe PIP joint contracture (>40°) is equivocal, as is the effect of patient adherence on outcome. Whilst total active extension deficit improved in some patients wearing a splint there were also deficits in composite finger flexion and hand function. The lack of data on the magnitude of this effect makes it difficult to interpret whether this is of clinical significance. There is a need for well designed controlled trials with proper randomisation to evaluate the short-term and long-term effectiveness of splinting following Dupuytren's surgery.

## Background

### Dupuytren's disease

Dupuytren's disease is a fibroproliferative disorder characterized by the development of nodules and contractile cords of the palmar fascia of the hand. Where the contractile cords cross the metacarpophalangeal (MCP) joint or the proximal interphalangeal (PIP) joint contractures of the digit into the palm occur. It is estimated that 20% of men in the UK over the age of 60 have Dupuytren's disease[1]. Women are affected later in life but have a similar prevalence to men in their eighth decade[2]. Treatment is indicated when the contracted finger interferes with the person's daily activities[3]. Surgical release of the contracted digit with or without excision of the affected tissue is the currently accepted intervention [1] with approximately 12,000 of these operations performed each year in the UK[4].

### Post-operative management

Hand therapy is advocated following Dupuytren's surgery with the overall aim to improve hand function[5]. The therapist uses a variety of techniques and modalities to promote wound healing, manage scar tissue, maximise finger extension and flexion and restore function[6]. Splinting is a commonly prescribed therapeutic modality designed to maximise the finger extension achieved from Dupuytren's surgery. Surveys of surgeons and therapists in the UK have identified that between 55–98% of respondents feel there is a role for splinting following Dupuytren's surgery [7-9]. Although a majority use splinting, there is a wide variation in how frequently it is applied. Salim [10] found that 14% of surgeons surveyed splinted following all operations, 19% usually splinted and 22% rarely did. There is also wide variation regarding position, force and duration of splinting[7,9]. Currently decisions regarding the indications for splinting and the parameters of the splint design and use are based on clinical experience and surgeon preference due to a lack of high quality evidence[6,8].

### Rationale for splint use

Scar contracture is a recognized complication following Dupuytren's surgery [11]. Prolonged low-load application of force is advocated as the most effective way to positively influence scar remodelling[12]. Controlling scar tissue with prolonged low load tension can prevent contracture of the MCP and PIP joints. Although exercises can deliver a prolonged low-load force, splinting has been identified as the most practical method to achieve the necessary time application [12] especially when used overnight.

A persistent PIP joint contracture is a known complication following Dupuytren's surgery[13]. Such contractures may be due to capsular tightness following prolonged PIP joint flexion from the Dupuytren's disease or due to complications from the operation itself, for example oedema restricting movement[4]. Splinting has been shown to be an effective treatment for both chronic (greater than six months duration) and acute (21 days to six months duration) PIP joint flexion contractures following PIP joint soft tissue injuries[13,14]. The reasoning for using splints after surgery for DD is that they provide a low-load continuous force which maintains the correction achieved intra-operatively and prevent contracture recurrence. Such splints are normally worn for 3 to 6 months as scar maturation and therefore the splints' effect on remodelling continues for this time period.

### Rationale for not using splints

On the other hand splinting is not a benign modality. Indiscriminate or inappropriate use of immobilization through splinting may result in joint stiffness, prolonged pain, oedema and subsequently reduced function[15]. Misapplication of force may also have unwanted effects on soft tissue for example attenuation of ligaments resulting in joint instability or overgrowth of scar tissue in response to increased stress[12]. It is also questionable after in vitro studies showed that stress may have an accelerative effect on the fibroproliferative process in Dupuytren's tissue[16]. From a practical perspective, splinting is time consuming and potentially expensive for the clinician. Patients may consider it inconvenient or even a nuisance to wear a splint for prolonged periods.

### The need for a systematic review investigating splinting following Duputyren's surgery

Surveys have highlighted the lack of consensus regarding indications for and parameters of splinting following Dupuytren's surgery and refer to the lack of good quality evidence[7,8]. Based on clinical experience and evidence extrapolated from other populations, many surgeons and therapists feel it is prudent to use a splint following surgery in some or all Dupuytren's patients. The purpose of this article is to identify and appraise available evidence regarding the effectiveness of splinting following Dupuytren's surgery. Such reviews provide a useful synthesis of evidence to date which may guide evidence-based clinical decisions and highlight the need for further primary research.

## Methods

A systematic review of the literature was undertaken to evaluate the quantity and quality of evidence regarding the clinical effectiveness of post-operative splinting after surgical release for DC in the hand.

### Search strategy

The electronic bibliographic databases Medline, AMED, CINAHL and EMBASE (from their inception to October 2007) were searched using the following terms: Dupuytren's contracture, splints or splinting. All titles and abstracts retrieved were read and assessed according to inclusion criteria. Full text was obtained for those articles which met the inclusion criteria or where it was impossible to assess these from the title and abstract. The references for each study which met the inclusion criteria were also scanned to identify any further studies not retrieved through the initial search. Additionally the *Journal of Hand Surgery *(British and European, and American Volumes), *Journal of Hand Therapy *and *British Journal of Hand Therapy *from 1990 to date were hand searched.

Inclusion criteria were:

• Prospective or retrospective, experimental, quasi-experimental or observational studies investigating the effectiveness of static or dynamic splints worn day and/or night-time for at least 6 weeks after surgery

• Studies reporting at least one of the following outcomes: individual joint or composite finger range of motion, hand function

Exclusion criteria:

• Studies primarily designed to investigate the effectiveness of different surgical techniques or approaches (for example fasciectomy versus dermofasciectomy) where splinting was used as part of the post-operative regimen

• Descriptive articles of splinting regimens and/or splint design and construction

• Studies of pre-operative splinting

• Non-English language articles

### Quality assessment

The focus of this systematic review was on the evaluation of effectiveness of post-operative splinting therefore randomised or non-randomised controlled trials needed to be included. Study design could have been used as a selection criterion or used to set a quality threshold, however the initial search highlighted a paucity of research and only one of the studies retrieved would have met this criterion. It was therefore decided to include the next best available evidence in the hierarchy of evidence, that is, observational studies and retrospective case series.

Each study was independently read and reviewed by both authors and assessed for methodological quality using the guidelines for evaluating the quality of intervention studies developed by McDermid[17]. It consists of 24 criteria which are scored as 0, 1 or 2 giving a maximum score of 48 (see legend to additional file 1 for criteria).

The scores from each reviewers were tabulated and compared. Where there was discrepancy the reviewers discussed the item until consensus on the score was reached.

## Results

**Figure 1:**
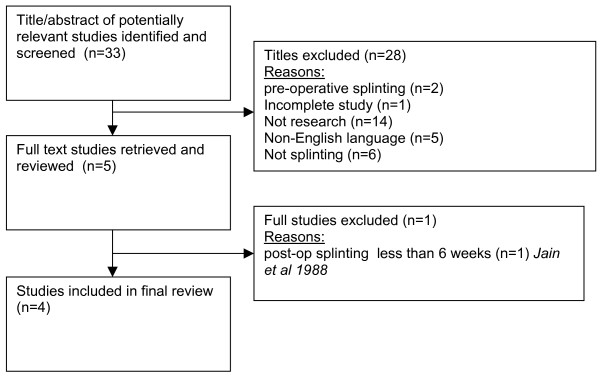
flowchart of studies retrieved through search and inclusion/exclusion criteria.

The initial search generated 33 references (excluding duplicates) for which the titles and abstracts were read to assess if they met inclusion criteria. 28 articles were excluded and the remaining 5 articles were obtained as full text and assessed by both authors. Jain et al's study [18] was subsequently excluded as the splinting intervention was less than 6 weeks leaving 4 studies available for review (see Figure 1).

There were no prospective, randomised, controlled trials and the 4 studies included one controlled trial without randomisation[19], one prospective observational study without control or comparator group[20], and one retrospective case review [21]. Evans et al's study [22] included retrospective case review as well as prospective comparison of two groups. Sample sizes ranged from 20 to 268 patients. The interventions studied included dynamic splinting, static splinting and the comparison of two positions in static splints. Mean follow up ranged from 9 weeks [22] to 2 years[20].

Tables provided in the additional files 2, 3, and 4 summarise the main characteristics and findings of each study.

### Quality assessment of studies

The final scores for each study using the MacDermid criteria [17] are presented in the additional file 1. Final quality scores for the 4 studies ranged from 17 to 22 points out of a total of a possible score of 48 points.

The level of agreement between the two reviewers for all four studies ranged from 54% to 66%. Any discrepancies between the reviewers related mostly to the interpretation of the criteria which once agreed resulted in consistent grading and no third reviewer was required to resolve any disagreements.

## Discussion

### The quality of evidence

The number of studies evaluating the effectiveness of post-operative splinting is small and the quality of the evidence is low. Two of the evaluation criteria were consistently not met by any of the studies, these were lack of randomisation to groups (question 5) and lack of sample size calculation (question 11). The low scores also reflect a lack of detail in proper reporting of results. Ebscov et al used proportion of patients with contractures and Rives et al only gave percentage improvement. No actual degrees of range of motion were reported making it also difficult to interpret the clinical significance. Glassey reported actual means and standard deviation for range of motion but not for self-reported hand function as measured by the Disabilities of the Arm, Shoulder and Hand Questionnaire (DASH[23]).

### Splinting interventions and effectiveness

Two studies investigated the effect of dynamic splinting[19,20]. Ebscov et al used a control group, though allocation to groups was not randomised and prone to allocation bias as only patients with rapidly recurring contracture of 25° or more were given a splint. This means that there are systematic differences between the two groups in terms of contracture severity. Those with recurrence are likely to have had a worse pre-op contracture and also likely to have had incomplete correction at surgery. Dias & Braybrooke [4] in an audit of Duputyren;s surgery found that recurrence of contracture was higher in those with greater initial deformity. In Ebscov et al's study the mean pre-operative contracture in the non-splint group was 23 degrees and 49 degrees in the splint group, respectively. The results at 9 months showed that a greater proportion of patients 12/15 (80%) in the splint group including those who met criteria for adequate wear (> 3 months) had an increased contracture of the PIPJ of 10 – 40 or > 40 degrees (see table 1a) compared to the no splint group (45%). Only 9/15 (60%) who were allocated to wear a splint but did not met the criteria for adequate wear had a PIP contracture of 10 – 40 or >40 degrees. It can be assumed that these two groups are comparable as they both had severe pre-op contractures of at least 25° and were allocated to receive a splint, yet compliance with the splint did not appear to benefit this group.

The study by Rives et al did not use a comparator group and all patients were allocated to receive a splint. Inclusion criteria were 'severe DC with PIPJ contracture ≥ 45° but excluding those with recurrent disease'. All patients were allocated to receive a splint however the results were analysed by compliance. Wear for 50% of time or longer was defined as compliant. The results from this group (n = 20) was compared to those in the non-compliant group (n = 7). Differences in percentage improvement of PIPJ extension between the compliant and non-compliant group were statistically significant at the 1, 3, 12 and 24 months follow-up (p < 0.05). Follow-up was the longest in this study and whilst the authors report statistically significant findings favouring the compliant group there is no indication of the magnitude of effect making it difficult to interpret the clinical significance of any differences. Moreover functional outcome such as the effect of reduced contracture on hand function was not measured. Rives's study highlights the important effect which patient compliance has on the efficacy of splints on final extension deficit, though this is contradicted by Ebscov's results which showed that a greater proportion of patients who had complied with splint wear had a recurrent contracture of 10–40 or >40 degrees.

Two studies investigated the effect of static splints. Glassey [21] conducted a retrospective review comparing the outcomes of patients who had been prescribed a static night splint (n = 21) worn for three months with those who had not (n = 10). Patients with a pre-operative MCP contracture only were excluded from the study. Use of a splint was determined by the surgeons' preference. Again bias is a concern due to the lack of randomised allocation and the small sample size. Baseline data indicated significant differences in age and occupational status between the groups. Other potential confounders such as severity of pre-operative contracture and bilateral presentation were not reported or included in the between group analysis. These factors are known to increase the risk of a persistent contracture [4,24] and would be known to the surgeon potentially influencing the prescription of a splint. The results at three months post-operatively found that function as assessed by the DASH and finger extension were significantly better in the non-splint group. Actual mean values and standard deviation for DASH scores were not provided so it is impossible to know if the difference in score is clinically meaningful or lies within the boundaries of measurement error. The splint group lost an average of 4.76° extension whilst the non-splint group gained an average of 13.75° (see additional file 3).

Evans et al [22] combined a retrospective case review with a prospective observational study investigating the effects of tension applied (TA) (n = 103) from splinting in the first three weeks following Dupuytren's surgery versus no tension applied (NTA) (n = 165). At three weeks, all patients were treated with the same night extension splint. All patients attending the clinic were included. The TA group was retrospectively studied with the prospective component investigating TA versus NTA. It is not described how the groups are allocated in the prospective component of the study but randomisation is not mentioned. The groups experienced similar outcomes in terms of range of motion however the NTA group required fewer therapy visits over a shorter period of time with fewer scar and flare symptoms (see additional file 4). The scales used to assess scar and flare have not been tested for reliability and validity therefore introducing doubt as to their value. The short follow-up period of five to ten weeks is also insufficient to judge the long term effects of the intervention as contractures can develop up to a year following a Dupuytren's operation[20].

### Outcomes assessed

All studies used MCPJ and or PIPJ range of motion (RoM) as the primary outcome measure. There were also differences in the way RoM was reported with some only measuring the degree of contracture or lack of MCP or PIPJ extension. It could be argued that a splint may assist in maintaining finger extension however anecdotal evidence suggest that patients often complain that after a night of wearing a splint they find it difficult to flex their digits or make a fist. Only Glassey [21] included total active extension and total active flexion. In her retrospective case review at 3 months patients without a splint had 20° more total flexion than the splint group, although this difference was not statistically significant.

Whilst degree of operative correction and hence the amount of joint extension is indisputably an important outcome it only reflects change at the level of impairment. Other outcomes including activities and participation were only addressed in Glassey's study through the inclusion of the DASH. Notably the DASH score in the non-splint group was significantly better than in the splint group, though actual mean DASH scores were not reported for either group making it difficult to interpret whether this difference was minimally clinically important at ≥12 points[25].

### Length of follow-up

The length of follow-up ranged from a few weeks to two years. Three of the four studies [19-21] used the splint for up to 6 months. It is debatable as to what is an appropriate endpoint to evaluate the effectiveness of splinting which also depends on what the primary outcome measure is. If recurrence of the contracture is the main criterion than outcome may need to be assessed no earlier than 1 year. On the other hand the effect of night splinting on loss of full composite finger flexion and hand function is likely to manifest itself sooner and possibly only during the period during which the splint is worn, that is up to 6 months, justifying a shorter follow-up period of 3 or 6 months.

### Limitations

Limiting this review to English language publications only may have lead to the exclusion of good quality studies, though the initial screening of titles and abstracts did not identify any RCTs in foreign language journals. The small number and heterogeneity of studies as well as the low methodological quality has limited the synthesis of findings and conclusions which can be drawn.

## Conclusion

This review identified only 4 studies providing low level evidence on the effects of static and dynamic post-operative splinting. None of the studies allocated patients to interventions randomly and therefore systematic differences between groups are likely to have biased results. The quality of the reporting was poor and due to the heterogeneity in splint types, duration of wear, outcomes and follow-up period it was not possible to pool the results. Whilst one study indicates that splinting results in fewer contractures especially when patients are compliant with wearing a splint another study did not support this, favouring the group who did not meet criteria for adequate wear, though follow-up for the latter group was also shorter (9 months). Although improved finger extension is undoubtedly an important outcome of long-term night splinting, composite finger flexion and hand function are also important parameters of the effect of splinting. Only one study included these as outcomes and the results indicate that patients allocated to wear a splint had lower total finger flexion and higher DASH scores (greater disability) at 3 months. These 'negative' effects may well disappear at 6 month or 1 year but no study to date has examined this.

Whilst the value of splints in delivering a low-load prolonged stretch to healing tissues after hand trauma and hand surgery has been well argued its effect on the diseased fascia in DD even after surgical excision remains unknown. Post-operative splinting is widely used and like many interventions in hand rehabilitation supported only by clinical reasoning and anecdotal evidence. The clinical effectiveness of long-term static night splinting on finger movement and hand function remains unproven and a properly randomized controlled trial is needed with a sufficient sample size to confer adequate power for detecting clinically important differences. The effect of different types of splints, duration and patient adherence need to be factored into future trials. Further work is also needed to establish the most appropriate primary and secondary outcomes and follow-up time in future studies of effectiveness.

## Competing interests

The authors declare that they have no competing interests.

## Authors' contributions

DL and CJH conceived the original idea, undertook the searches and review of articles and drafted the manuscript. Both authors have read and approved the final manuscript.

## Pre-publication history

The pre-publication history for this paper can be accessed here:



## Supplementary Material

Additional file 1 Results of evaluation of quality of intervention studies using McDermid (2004) criteria. Click here for file

Additional file 2 Studies investigating effectiveness of dynamic splints. Click here for file

Additional file 3 Studies investigating effectiveness of static splints. Click here for file

Additional file 4 Studies comparing effectiveness of different static splints. Click here for file
