# Supplementary material for: Clinical effectiveness of post-operative splinting after surgical release of Dupuytren's contracture: a systematic review
Source: BMC Musculoskelet Disord. 2008 Jul 21;9:104. doi: 10.1186/1471-2474-9-104 (PMC2518149; doi:10.1186/1471-2474-9-104)
Supplement: Additional file 1 — Results of evaluation of quality of intervention studies using McDermid (2004) criteria. [file 1471-2474-9-104-S1.doc]

**Table 4: Results of evaluation of quality of intervention studies using McDermid (2004) criteria**

| **study** | **Background and design** | | | | | | | | **subjects** | | | | **intervention** | | | **outcomes** | | | **Analysis and recommendations** | | | | | |  |
| --- | --- | --- | --- | --- | --- | --- | --- | --- | --- | --- | --- | --- | --- | --- | --- | --- | --- | --- | --- | --- | --- | --- | --- | --- | --- |
|  | **1** | **2** | **3** | **4** | **5** | **6** | **7** | **8** | **9** | **10** | **11** | **12** | **13** | **14** | **15** | **16** | **17** | **18** | **19** | **20** | **21** | **22** | **23** | **24** | **Total** |
| Ebscov et al | 1 | 1 | 2 | 2 | 0 | 1 | 1 | 0 | 1 | 1 | 0 | 2 | 1 | 0 | 2 | 1 | 0 | 1 | 1 | 0 | 0 | 1 | 0 | 2 | 21 |
| Evans et al | 1 | 1 | 0 | 0 | 0 | 0 | 1 | 0 | 1 | 0 | 0 | 1 | 1 | 1 | 2 | 0 | 1 | 1 | 1 | 1 | 1 | 2 | 1 | 0 | 17 |
| Glassey | 1 | 1 | 2 | 0 | 0 | 1 | 1 | 0 | 1 | 0 | 0 | 2 | 1 | 1 | 2 | 2 | 1 | 0 | 2 | 0 | 1 | 1 | 0 | 2 | 22 |
| Rives et al | 1 | 0 | 2 | 2 | 0 | 1 | 1 | 0 | 1 | 2 | 0 | 2 | 1 | 1 | 0 | 1 | 1 | 2 | 1 | 0 | 1 | 1 | 0 | 0 | 21 |

A score of 2 means that all criteria have been met, 1= partially met and 0 = criteria not met for the question.

The evaluation criteria (for further guidelines on these criteria see Macdermid 2004, page 114-117)

1. was the relevant background work cited to establish a foundation for the research question?
2. was a comparison group used?
3. was patient status at more than one time point considered?
4. was data collection performed prospectively?
5. were patients randomised to groups?
6. were patients blinded to the extent possible?
7. were treatment providers blinded to the extent possible?
8. was an independent evaluator used to administer outcome measures?
9. did sampling procedures minimise sampling/selection bias?
10. were inclusion/exclusion criteria defined?
11. was an appropriate enrolment obtained
12. was appropriate retention/follow-up obtained?
13. was the intervention applied according to established principles?
14. were biases due to the treatment provider minimised?
15. was the intervention compared with an appropriate comparator?
16. was an appropriate primary outcome defined?
17. were appropriate secondary outcomes considered?
18. was an appropriate follow-up period incorporated?
19. was an appropriate statistical test performed to indicate differences related to the intervention?
20. was it established that the study had sufficient power to identify treatment effects?
21. was the size and significance of the effects reported?
22. were missing data accounted for and considered in the analysis?
23. were clinical and practical significance considered in interpreting results?
24. were the conclusions/ clinical recommendations supported by the study objectives, analysis and results?
