# Supplementary material for: Clinical effectiveness of post-operative splinting after surgical release of Dupuytren's contracture: a systematic review
Source: BMC Musculoskelet Disord. 2008 Jul 21;9:104. doi: 10.1186/1471-2474-9-104 (PMC2518149; doi:10.1186/1471-2474-9-104)
Supplement: Additional file 2 — Studies investigating effectiveness of dynamic splints. [file 1471-2474-9-104-S2.doc]

**Table 1: Studies investigating effectiveness of dynamic splints**

| **Author, date** | **Study design** | **Patients (n= )** | **Surgical procedure** | **Experimental intervention - splint** | **Duration and frequency of splint** | **control** | **Length of follow-up**  **Outcomes assessed** | **Results** |
| --- | --- | --- | --- | --- | --- | --- | --- | --- |
| Ebskov et al 2000 | Prospective, non- randomized controlled trial | Inclusions: advanced DD with ≥ 2 rays affected (n=69)  Age, gender not reported | Open palm technique and radical fasciectomy  + PIP capsulectomy (n=27), K-wire (n=3) | Criteria: rapid recurrence or 25º residual contracture  Dorsal dynamic extension splint (200g force) | Commenced 2-3/52 post-op  Night wear for 6/12 | No splint | Pre-operatively, 3/52 and 9/12 post-op  i) Use of splint: daily at least 3/12 = adequate use, less than above = inadequate, no splint  ii) range of motion: change of ≤10º =same, ± 10-40º, >40º  iii) mean (SD) RoM | See Table 1a  Higher recurrence of contracture in PIPJ in ‘adequate’ splint group, however criteria for splint group were rapid recurrence or residual contracture of 25º |
| Rives et al 1992 | Prospective observational study | Inclusions: Severe DC ≥45º  Exclusions: digits with recurence  n=23 (PIP joints in 20 patients)  75% male  Age: mean 60 (44-76)  PIPJ contractures: mean=66º (45-110º) | McCash open-palm with Bruner zig-zag incisions  Capsular release in 18 digits | Dorsal dynamic splint (DS) with 200gms dynamic extension force to PIPJ | DS worn daytime and night  at 4/52 replaced with static splint (SS) for night and DS worn in day  from 8/52 to 6./12 DS decreased to 2 hours 3 times per day, SS at night | n/a | Mean follow-up 2 years (1 to 3 ½ years)  Compliance: patient-report, <50% adherence classified as non-compliant (n=13)  % improvement in PIPJ– measurement of joint angle tool and procedure not described | See Table 1 b  Percent improvement was higher with longer splint wear mean improvement 59% (compliant) and 25% (non-compliant)  Compliance was only factor which significantly affected outcome  Severity of contracture, digit affected and capsular release did not significantly affect outcome |

**Table 1a: Ebskov et al (2000) results with calculated percentages in each group at 9 months follow-up**

| **contracture** | **MCPJ** | | | **PIPJ** | | |
| --- | --- | --- | --- | --- | --- | --- |
|  | **Adequate splinting**  **n=14** | **Inadequate splinting**  **n=15** | **No splint**  **n=23** | **Adequate splinting**  **n=15** | **Inadequate splinting**  **n=15** | **No splint**  **n=24** |
| **≤10º** | 8 (57%) | 9 (60%) | 19 (82.6%) | 3 (20%) | 5 (33.3%) | 11 (46%) |
| **Increased 10-40º** | 2 (14.3%) | 3 (20%) | 3 (13%) | 10 (66.6%) | 7 (46.6%) | 9 (37.5) |
| **Increased > 40º** | 3 (21.4%) | 1 (6.6%) | 0 | 2 (13.3%) | 2 (13.3%) | 2 (8.3%) |
| **Decreased 10-40º** | 1 (7%) | 0 | 1 (4.3%) | 0 | 1 (6.6%) | 2 (8.3%) |
| **Decreased > 40º** | 0 | 2 (13.3%) | 0 | 0 | 0 | 0 |
| **Total** | 14 | 15 | 23 | 15 | 15 | 24 |

**Table 1b: Rives et al 1992: PIPJ extension measured as % improvement**

|  | **1 month** | **3 months** | **12 months** | **24 months** |
| --- | --- | --- | --- | --- |
| **Compliant with splint (n=13)** | 94% | 80% | 78% | 69% |
| **Discontinued splint at 1.5 to 3 months** | 82% | 58% | 25% | 25% |
| **discontinued splint in first few weeks** | 47% | 23% | 25% | 33% |
