# Supplementary material for: Clinical effectiveness of post-operative splinting after surgical release of Dupuytren's contracture: a systematic review
Source: BMC Musculoskelet Disord. 2008 Jul 21;9:104. doi: 10.1186/1471-2474-9-104 (PMC2518149; doi:10.1186/1471-2474-9-104)
Supplement: Additional file 3 — Studies investigating effectiveness of static splints. [file 1471-2474-9-104-S3.doc]

**Table 2: Studies investigating effectiveness of static splints**

| **Author, date** | **Study design** | **Patients (n= )** | **Surgical procedure** | **Experimental intervention - splint** | **Duration and frequency of splint** | **control** | **Length of follow-up**  **Outcomes assessed** | **Results** |
| --- | --- | --- | --- | --- | --- | --- | --- | --- |
| Glassey 2001 | Retrospective case review | Surgery for contractures of MCPJ and PIPJ  Splint group (n=21) age: 68.8 (±7.6)  no splint group (n=10) age: 58.5(±13.2)  81% male | fasciectomy | Static volar hand based thermoplastic night splint  Only given to those patients judged to require splint due to loss of post-op extension or surgeon preference | Applied at first dressing change and worn night-time for 3 months | No splint – those where it was not deemed necessary or surgeon preference | @ 3/12  RoM: Total active flexion (TAF), Total lack of active extension (TLAE) Total active movement (TAM) according to ASSH assessed with goniometer  Power grip: Jamar  Pain: VAS  Hand function: DASH | No significant baseline differences except age  at 3/12:  magnitude of effect not given only p-values  DASH better in no splint group (p=0.01)  grip: NS pain: NS  TLAE: Splint -4.76 (±21.3), No Splint 13.75 (±25.12) (p=0.04)  TAF: Splint 42.62 (±30.9), No Splint 62.25 (±32.5) p=0.11  TAM: Splint 35.95 (±40) No Splint 74.5 (±36) (p=0.02) |
