# Supplementary material for: Clinical effectiveness of post-operative splinting after surgical release of Dupuytren's contracture: a systematic review
Source: BMC Musculoskelet Disord. 2008 Jul 21;9:104. doi: 10.1186/1471-2474-9-104 (PMC2518149; doi:10.1186/1471-2474-9-104)
Supplement: Additional file 4 — Studies comparing effectiveness of different static splints. [file 1471-2474-9-104-S4.doc]

**Table 3: Studies comparing effectiveness of different static splints**

| **Author, date** | **Study design** | **Patients (n= )** | **Surgical procedure** | **Experimental intervention - splint** | **Duration and frequency of splint** | **control** | **Length of follow-up**  **Outcomes assessed** | **Results** |
| --- | --- | --- | --- | --- | --- | --- | --- | --- |
| Evans et al 2002 | a)retrospect case review  b) prospect observational study | DC treated by fasciectomy from 1983-99  Tension applied (TA) n=103  age: 67.15 + 8.91  No tension applied (NTA) n=165  age: 69.33 + 6.78  76% male | Fasciectomy performed by 49 different surgeons | TA – MCPJ 0-20º and PIP extended  NTA – 40-45º MCPJ and PIP extended in weeks 1 to 3, then replaced with MCP extended | TA splint – intermittent day use + night-time  NTA splint – with dorsal blocking of MCPJ for 24 hours in weeks 1-3, PIPJ extension delayed till day 7 to 20  then night-time only  duration – not specified | n/a | Mean follow-up: TA=68 days, NTA=36 days  Wound flare – grade 0 - 2  Scar pliability – grade 0 - 2  MCP and PIPJ RoM – method not described  No of therapy visits, length of therapy | NTA – fewer scar complications, less flare and fewer therapy visits  MCP/PIPJ contracture: TA = 11º NTA = 4º  Days of therapy: TA = 68, NTA = 37 (p<0.01)  No of therapy visits: TA = 20, NTA = 13 (p<0.01) |
